# Molecular Analysis of Atypical Family 18 Chitinase from Fujian Oyster *Crassostrea angulata* and Its Physiological Role in the Digestive System

**DOI:** 10.1371/journal.pone.0129261

**Published:** 2015-06-05

**Authors:** Bingye Yang, Mingming Zhang, Lingling Li, Fei Pu, Weiwei You, Caihuan Ke

**Affiliations:** 1 Xiamen Medical College, Xiamen, 361008, PR China; 2 State Key Laboratory of Marine Environmental Science, Xiamen University, Xiamen, 361005, PR China; 3 College of Ocean and Earth Science, Xiamen University, Xiamen, 361005, PR China; 4 College of Life Science, Xiamen University, Xiamen, 361005, PR China; Zhejiang University, CHINA

## Abstract

Chitinolytic enzymes have an important physiological significance in immune and digestive systems in plants and animals, but chitinase has not been identified as having a role in the digestive system in molluscan. In our study, a novel chitinase homologue, named Ca-Chit, has been cloned and characterized as the oyster *Crassostrea angulate*. The 3998bp full-length cDNA of *Ca*-Chit consisted of 23bp 5-UTR, 3288 ORF and 688bp 3-UTR. The deduced amino acids sequence shares homologue with the chitinase of family 18. The molecular weight of the protein was predicted to be 119.389 kDa, with a pI of 6.74. The *Ca*-Chit protein was a modular enzyme composed of a glycosyl hydrolase family 18 domain, threonine-rich region profile and a putative membrane anchor domain. Gene expression profiles monitored by quantitative RT-PCR in different adult tissues showed that the mRNA of *Ca*-Chit expressed markedly higher visceral mass than any other tissues. The results of the whole mount in-situ hybridization displayed that *Ca*-Chit starts to express the visceral mass of D-veliger larvae and then the digestive gland forms a crystalline structure during larval development. Furthermore, the adult oysters challenged by starvation indicated that the *Ca*-Chit expression would be regulated by feed. All the observations made suggest that *Ca*-Chit plays an important role in the digestive system of the oyster, *Crassostrea angulate*.

## Introduction

Chitin belongs to a nitrogen-containing polysaccharide bio-polymer and is found widely spread across the earth. The Chitin polymer has been found as a structural component in the shell of crustaceans, as seen in shrimp, crabs, insects, the organs of invertebrate, the cell wall of fungi and some algae. Chitin is one of the most abundant carbohydrates present in the marine environment and the second most abundant bio-polymer on earth, next to cellulose [1]. Annual biosynthesis estimates range from 1010 to 1011 metric tons [2]. Chitin plays an important role in the ocean’s food chain and carbon cycle because of its ubiquitous and extensive presence in marine ecosystems.

The Fujian oyster *crassostrea angulata* is an important edible marine bivalve, which has been harvested from the wild and cultivated for centuries. The growth and development of oysters is closely related to its digestive system. Oyster belong to bivalves take algae which contain chitin as main food, degradation of chitin-containing structures requires chitinases, so which suggested that the digestive gland of organism in bivalves probably secrete chitinases when the algae as food enter into their digestive system. The bivalve digestive system is comprised of a complex stomach and associated structures, but an otherwise simple intestine, actually in the bivalve *Scrobicularia plana*, Chitinase had been found in the gastric shield of digestive gland by histochemiscal technique [3], and also some chitinases are detected in the digestive tract of mammalians [4,5] and mollusks [6,7], But until now, there is no gene expression analysis of chitinase reported in bivalves.

The crystalline style is also an important part of digestive gland, which is present in nearly all bivalve mollusks and some plant-eating Prosobranchia [8,9]. The crystalline style is known to undergo cyclical changes in size and/or occurrence in many species [10–13], which is a distinct formation with a cylindrical shape, concentric layered structure, jelly consistency, and complex chemical composition [14,15]. The crystalline style has many functions, but the main digestive function which enhances the digestion of food particles was beyond a doubt. The crystalline structure is the principal organ of extracellular digestion, which is rotated in its sac by cilia, which head projects into the stomach and grinds against part of the chitinous gastric shield lined stomach wall. Chitinase activity has been detected in the crystalline structure of the American oyster *Crassostrea virginica* [16]. In other respects, two genes encoding the chitinase protein and chitinase-like protein had been characterized with their cDNAs from the oyster *Crassostrea gigas*, but both of them functioned on the immunity [17,18]. While the chitinase activities were measured by the crystalline structure and the digestive tract of several molluscs [19], no chitinase gene of family 18 has been identified at the molecular level in the Bivalves.

In this study, a chitinase gene was characterized and the full cDNA sequence was identified in *Crassostrea angulata*. Tissue distribution and the temporal spatial pattern of expression during larval development were established by real time PCR and in situ hybridization. We also examined the gene chitinase expression of the adult oyster after starvation and food treatment. Our results provided insight into the study of chitinase and its effects on the digestive system of oysters at the molecular level.

## Materials and Methods

### Sample collection and larva culture

The oyster *Crassostrea angulata* is not a protected species, and collections were only made from public access areas, no specific permits were required to collect this species in Wuguan aquaculture farm of xiamen city in Fujian (GPS coordinates: 24.52,118.06). The adult oysters, *C*. *angulata*, were collected from the Xiamen coast and dissected to obtain different tissues, including gills, visceral mass, female gonad, male gonad, hemocytes, mantle, muscle and palps. There are three parallel samples for each tissue. The samples were washed with 1×PBS (phosphate-buffered saline), frozen in liquid nitrogen and stored at -80°C until processed. Larvae culture of C. *angulata* was conducted as previously described [20]. For whole mount in-situ hybridization (WMISH), trochophore were collected and then fixed directly in 4% paraformaldehyde overnight at 4°C. The larvae were first anesthetized by gradually adding MgCl_2_ solution to the seawater and then collected for fixation. The trochophore were dehydrated in gradient methanol and stored in 100% methanol at −20°C.

### Adult oyster starvation challenge

The adult oysters were cultured in filtered seawater between a temperature of 25°C to 26°C, and salinity of 25.0 to 27.0 without feeding. After 6 days and 7 days the adults were dissected to obtain the visceral mass, while the other adults were fed with *Platymonas subcordiformis*, *Dicrateria zhanjiangenis* and soy milk. This was followed by the dissection of the fed adults at 2h, 9h and 48h to obtain the visceral mass at various times. These visceral masses were washed with 1×PBS, frozen in liquid nitrogen and stored at -80°C until processed.

### RNA Extraction and the first-strand synthesis

The total RNA of each sample was extracted with the RNAzol RNA isolation kit (Biotecx, Houston, TX, USA) according to the manufacturer's instructions. RNA integrity was checked by separating on a 1% formaldehyde-denatured agarose gel and staining with ethidium bromide. The quantity of RNA was determined by measuring OD 260nm with the NanoDrop ND-1000 UV-Visible Spectrophotometer (ThermoScientific, USA). The total RNA was reverse-transcribed into cDNA as described previously [20]. The first strand of the cDNA was synthesized and used as the template for further PCR analysis.

### Molecular cloning of the chitinase-coding gene and sequence analysis

In order to clone the full sequence of the chitinase-coding gene, 5′-RACE and 3′-RACE tests were conducted separately using the Takara 5′-full RACE and 3′-full RACE cDNA Amplification Kit (Takara, Dalian, China) according to the manufacturer's instructions. The processes involved were described in detail previously, except for the primers [21]. The specific primers related to *Ca*-chit were designed based on the nucleotide sequence of chitinase cDNA fragments, which were obtained from the *C*. *angulata* transcriptome library and sequenced through 454 sequencing technology [22]. The primers for the 5′-RACE and 3′-RACE are shown in Table 1. In this study the chitinase-coding gene from *C*. *angulata* was designated as *Ca*-Chit. The full sequence of *Ca*-Chit has been submitted to Genbank (GenBank accession number: KJ438173).

**Table 1:** List of primers sequences used in this study.

Name	Experiment	Sequence(5'-3')
*Ca*-Chit-F	Confirm-PCR	CACCTGATCTACGCCTTTG
*Ca*-Chit-R	Confirm-PCR	CCCTTAGTTGACCCTGTT
*Ca*-Chit-F1	RACE	CCCAAATGCTCCGCTGAAAG
*Ca*-Chit-F2	RACE	AATCAACATGATGGCGTATG
*Ca*-Chit-R1	RACE	TCACTGCCCACTCGTCAT
*Ca*-Chit-R2	RACE	CGGGGTATTCCCAGTCC
18S-F	qRT-PCR	CGGGG AGGTA GTGACGAA
18S-R	qRT-PCR	ACCAG ACTTG CCCTC CAA
EF-1α-F	qRT-PCR	ACCACCCTGGTGAGATCAAG
EF-1α-R	qRT-PCR	ACGACGATCGCATTTCTCTT
*Ca*-Chit-F2	qRT-PCR	TCTATGCTGACCCTAACTCTT
*Ca*-Chit-R2	qRT-PCR	TGTTTGTTGTATTTGTGGC
*Ca*-Chit-F3	WISH	CCCAAATGCTCCGCTGAAAG
*Ca*-Chit-R3	WISH	GAATCCAATGGCGAACACCC

To confirm the accuracy of the sequence of *Ca*-Chit through the RACE, a pair of gene-specific primers, *Ca*-Chit F1 and *Ca*-Chit R1, were used for amplifying *Ca*-Chit cDNAs with polymerase Ex Taq (Takara, China), according to the following conditions: denaturation at 94°C for 5 min, followed by 31 cycles at 94°C for 30 s, 53°C for 30 s, and 72°C for 90 s. As an extension, a final step was conducted at 72°C for 10 min. The purified PCR products were cloned into a pMD-19T Vector, transmitted into competent cells of DH5α, plated flat on LB-Agar and sequenced in both directions as eight independent clones.

The entire nucleotide sequence was analyzed using the BLAST program available from the National Center for Biotechnology Information (http://www.ncbi.nlm.nih.gov/). DNAMAN (DNAMAN Lynnon Biosoft, Santa Clara, USA) was used to identify its encoding protein. The transmembrane domains of the protein sequence were predicted by the TMHMM Server (http://www.cbs.dtu.dk/services/TMHMM). Prosite Server (http://expasy.org/prosite/) was used to predict the functional alleles of the gene. Amino acid sequences were aligned using ClustalX (http://www.clustal.org/), while the phylogenetic tree was carried out in the Mega 4.1 program by using the neighbor-joining method of clustering, based on a PAM Matrix. The Bootstrap value was computed over 1,000 replications.

### *Ca*-Chit mRNA expression analysis

Quantitative RT-PCR was used to quantify changes in gene expression of the different tissues and the visceral mass in adults during the starvation experiment. There are three oysters in each group. The process of quantitative RT-PCR was described in detail previously [21]. The RT- qPCR reactions were conducted on ABI7500FAST. All samples were run in parallel with the housekeeping gene, 18S rRNA, with an elongation factor-1α (EF-1α). Annealing curves were performed to ensure the absence of primer dimers. Amplification products were also electrophoresed on 1% agarose gels to ensure that one single bandwas generated for each of the tested genes. The relative expression levels of target genes were calculated using a two-standard-curve method using the following formula N = 2^−△Ct [chit-(EF-1+α18S)/2]^ [23]. Standard curves for both the target gene and endogenous control gene were generated from 10-fold dilutions of cDNA samples to determine primer efficiency. Relative ratios of *Ca*-chit to EF-1α and 18s RNA mRNA in each samples were calculated based on the Ct value and the standard curve of the gene. The primers for Real-time qPCR have been shown in Table 1. The data represents the mean values of three biological replicates. Data of competitive real-time PCR analysis subjected to one-way analysis of variance (ANOVA) and followed by a multiple comparison test by using the LSD-t test to determine the difference in mean values was used with the SPSS software. The *P* value for significance was set at *P* ≤0.05.

### Whole-mount in-situ hybridization

Antisense and sense digoxigenin-labeled cRNA probes were synthesized with a DIG-RNA labeling Kit (Roche). A PCR fragment related to *Ca*-Chit was inserted into the PMD-19T PGEM-T EASY Vector (Promega), followed by the transformation of the litigation mixture into DH5α competent cells, and followed by sequencing. A plasmid was used as the template to amplify the *Ca*-Chit cDNA fragment, which was used as the template to be subjected to in vitro transcription. Riboprobes were synthesized by transcribing with T7 RNA polymerase and Digoxigenin-11-UTP (Roche). The whole-mount in-situ hybridization (WISH) procedure used for spatial expression analysis was based on the protocol used in ascidian [24], with some modifications. The process of WISH was described in detail previously [20]. Images were taken with a digital camera (Olympus DP71) of the fluorescence light microscope (Olympus BX51). Digital photographs were imported into Adobe Photoshop CS, where they were cropped and the brightness and contrast were optimized

## Results

### Isolation and sequence analysis of *Ca*-Chit full-length cDNA

**Fig 1:**
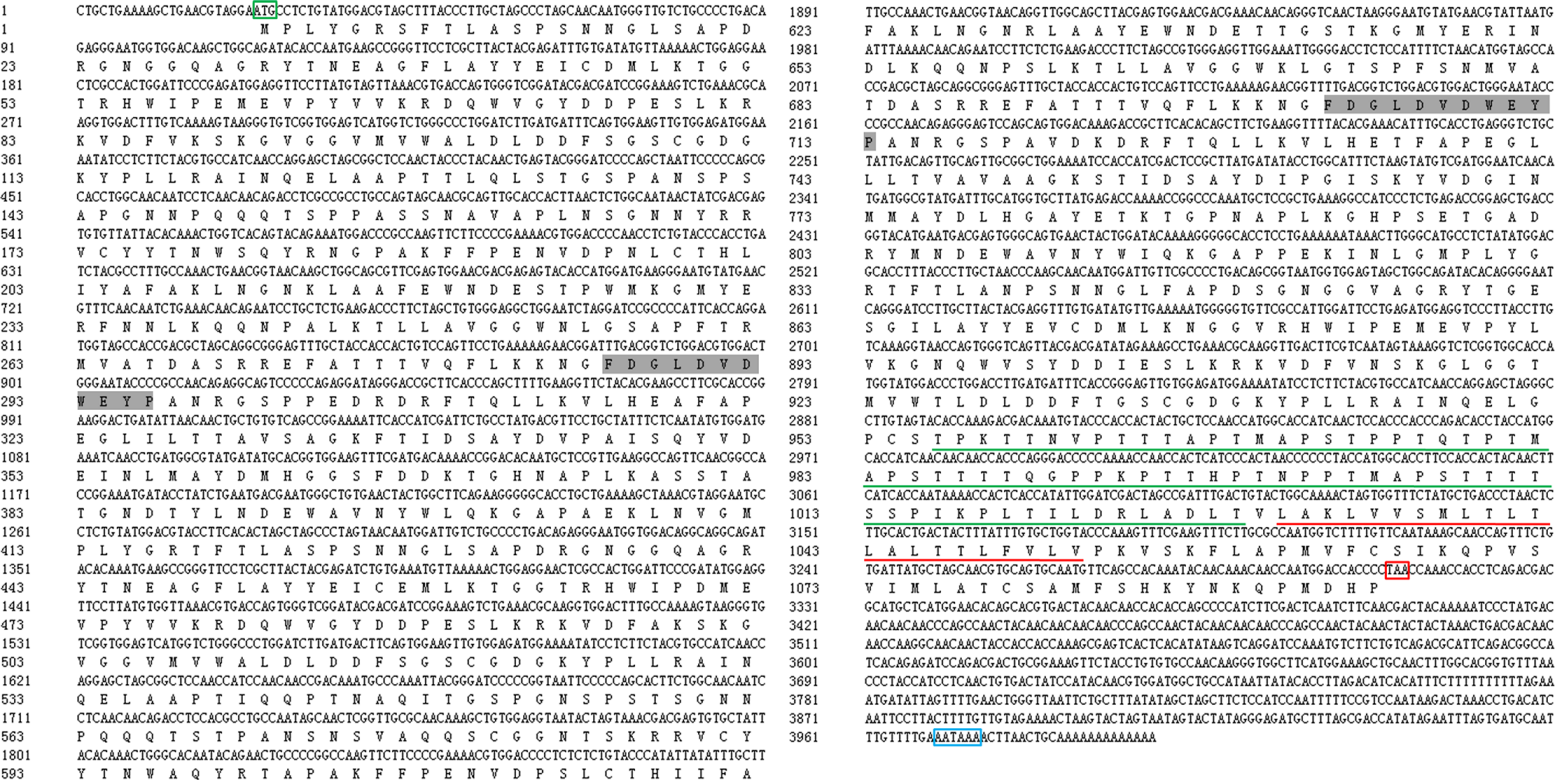
*Ca*-Chit cDNA and deduced amino acid sequences (GenBank accession number KJ438173) from *C*. *angulata*. The start and stop codons are boxed in green and red. The polyadenylation signal is boxed in blue. Amino acids corresponding to the active centre are marked in gray. The threonine rich domain and member anchor region are respectively underlined in green and red.

The cDNA fragments obtained from the *C*. *angulata* transcriptome library shared similarities with the amino acid sequence of members of the GH18 family. Subsequently, through 5′-full RACE and 3′-full RACE we obtained the full-length cDNA. Sequence analysis of cloned *Ca*-chit indicated that the 3998bp, full-length cDNA of *Ca*-Chit consisted of 23bp 5-UTR and 688bp, Open reading Frame starting with an ATG at position 24 and ending with a TAA at position 3311, and consisted of 3288 nucleotides encoding a protein of 1095 amino acid residues (Fig 1). The molecular weight of the deduced protein was predicted to be 119.389 kDa, with a pI of 6.74. The mature protein contains fourteen potential recognition sites for N-myristoylation, sixteen sites for casein kinase II phosphorylation, twelve sites for PKC phosphorylation, two sites for tyrosine kinase phosphorylation, and three sites for N-linked glycosylation [25,26]. The *Ca*-Chit protein was a modular enzyme composed of a glycosyl hydrolase family 18 domain with a threonine-rich region profile and putative membrane anchor domain.

**Fig 2:**
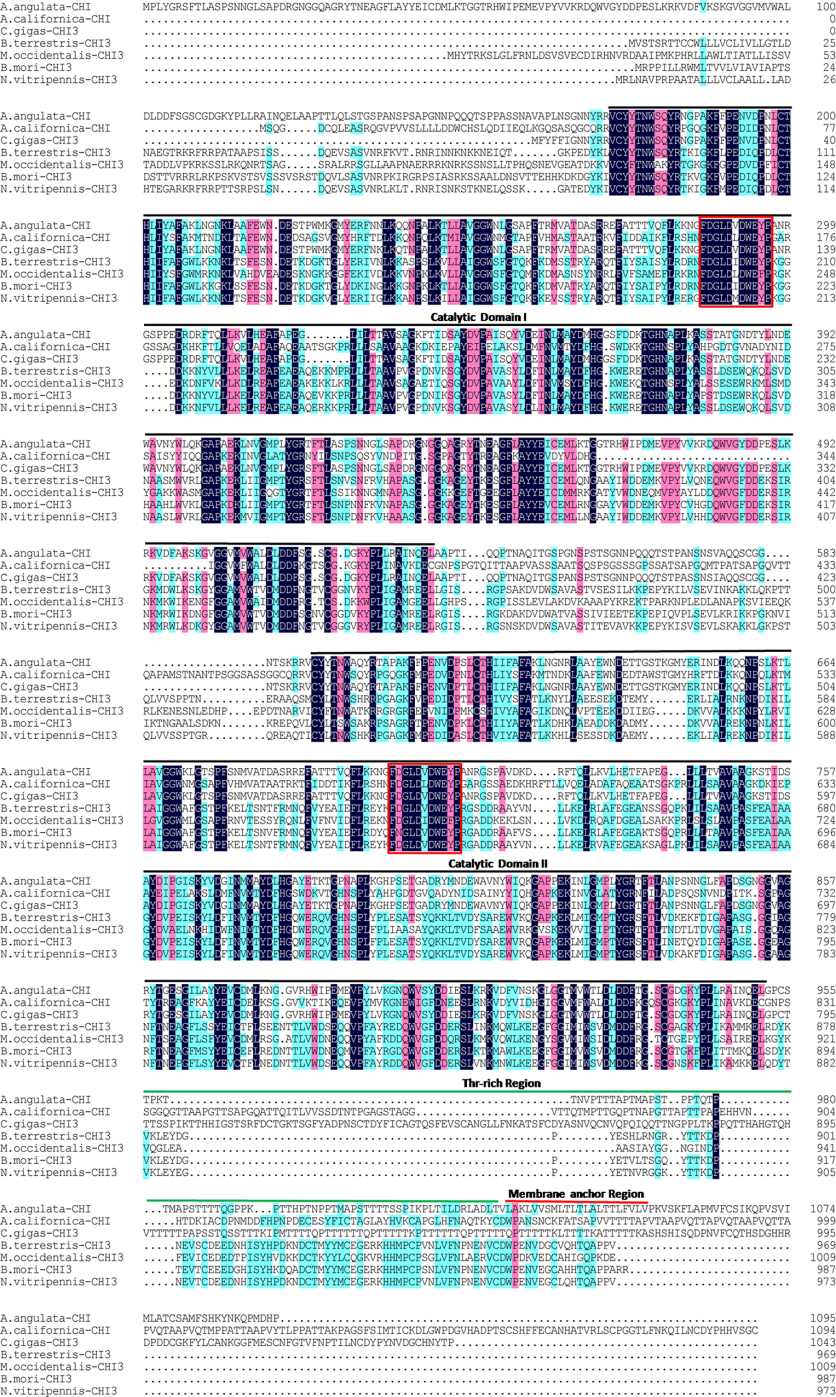
Multiple alignment of amino acid sequences of *Ca*-Chit with members of the GH18 family. The amino acid boxed in black indicates conservation of identical residues in all sequences. The amino acid boxed in pink indicates conservation of residues with above 75% consistency. The black line delimit two glycosyl hydrolase family 18 domains, two active centres are indicated in red boxes. The threonine rich domain and member anchor region are respectively uplined in green and red. Dots represent indels.

**Fig 3:**
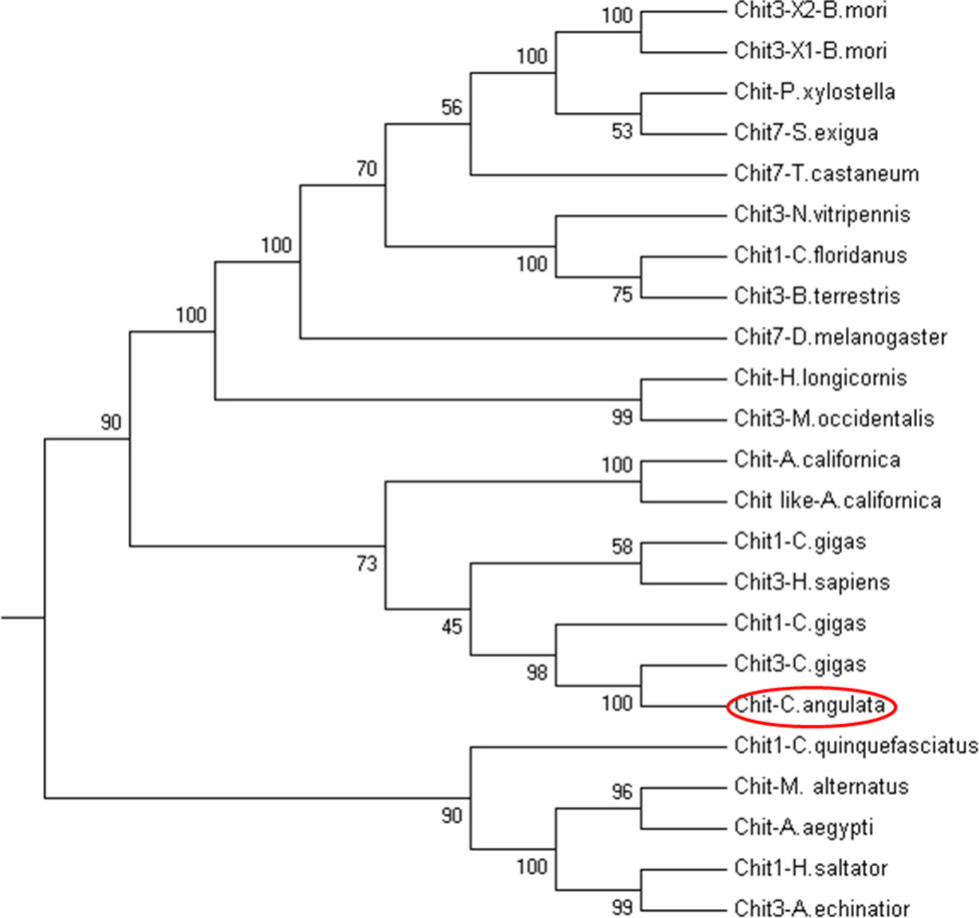
Phylogenetic tree analysis of *Ca*-Chit from *C*. *angulata* with members of the GH18 family using MEGA 4.0. The amino acid sequence alignment was carried out by ClustalX program, and the dendrogram was constructed by the neighbor-joining method of clustering based on a PAM Matrix. Bootstrap values were computed over 1,000 replications. The Genbank accession numbers are as follows: *Bombyx mori*, XP_004922005.1; *Bombyx mori*, XP_004922004.1; *Plutella xylostella*, AFI55112.1; *Spodoptera exigua*, AFM38213.1; *Tribolium castaneum*, NP_001036035.1; *Nasonia vitripennis*, XP_001604515.2; *Bombus terrestris*, XP_003400489.1;*Camponotus floridanus*, EFN67257.1; *Haemaphysalis longicornis*, BAC06447.1;*Drosophila melanogaster*, NP_647768.3; *Metaseiulus occidentalis*, XP_003741167.1;Aplysia californica, XP_005104423.1; *Aplysia californica*, XP_005104424.1;*Crassostrea gigas*, EKC38805.1; *Homo sapiens*, AAX54833.1; *Crassostrea gigas*, EKC38802.1; *Crassostrea gigas*, EKC38804.1; *Culex quinquefasciatus*, XP_001867701.1; *Monochamus alternates*, BAG13449.1; *Aedes aegypti*, AAB81850.1; *Harpegnathos saltator*, EFN79784.1; *Acromyrmex echinatior*, EGI58538.1.

A protein BLAST homology search [27] revealed significant identity with putative and actual genes encoding chitinases and the chitinase-like protein of the GH18 family. The deduced amino acids of *Ca*-Chit shared many similarities with the chitinase of oyster *Crassostrea gigas* (67.58%), sea hare *Aplysia californica*(33.74%), silkworm *Bombyx mori* (33.21%) and the parasitoid *Nasonia vitripennis* (31.76%). Optimal alignment of *Ca*-Chit with some of these homologous proteins revealed two putative, catalytic domains at the N-terminus of the protein which included two active centres (Fig 2). Phylogenetic tree analysis also demonstrated that it is relatively more closely to molluscan chitinase 3 (Fig 3).

### Tissue distribution of *Ca*-chit mRNA

**Fig 4:**
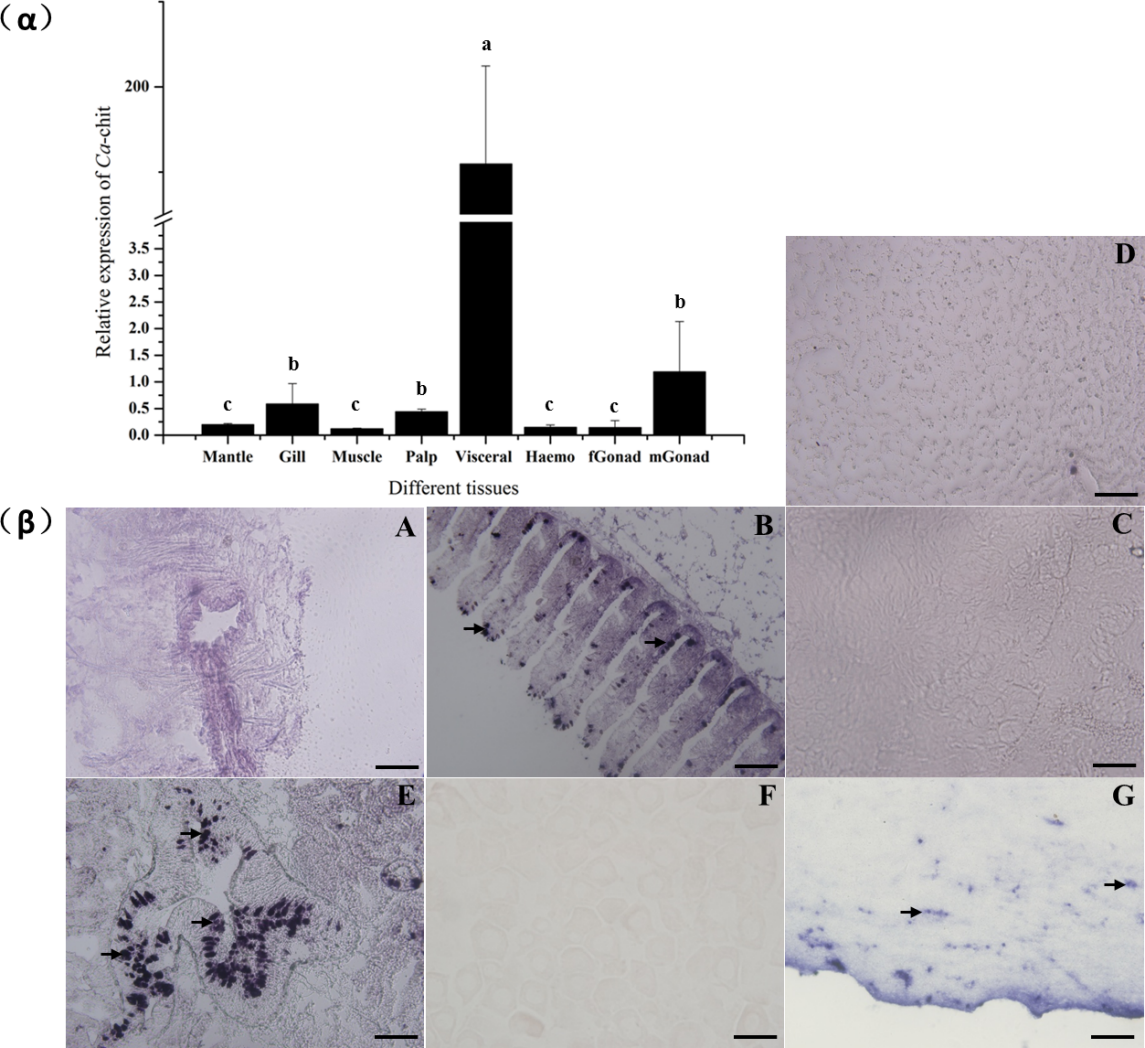
Expression analysis of *Ca*-Chit mRNA transcripts in different tissues using qRT-PCR (α) and in situ hybridization (β). Each bar represents the mean ± SD of three replicates from three oysters. Data with significant difference between each other at *P<0*.*05* are indicated by different letters. A: mantle; B: gill; C: muscle, D: palps; E: visceral mass; F: female gonad; 5, G: male gonad; Signals were labelled with arrow. The scale of black bar is 100 um.

To gain insight into the possible physiological functions of *Ca*-chit, tissue-specific expressions of *Ca*-chit were analyzed using quantitative RT-PCR and in situ hybridization in various tissues of normal *C*. *angulata*. As shown in Fig 4α, *Ca*-Chit mRNA was highly expressed in visceral mass, and *Ca*-Chit mRNA levels of other tissues was at least 100-fold less than the level in visceral mass (*p<0*.*05*), and the results in Fig 4β showed that *Ca*-Chit mRNA was localized in the apical lamina of digestive lumens, but interestingly, week sensitivity of detection was also found in gills and male gonads which is consistent with the results of qRT-PCR. The visceral mass is the main digestive system of oysters, which indicates that *Ca*-Chit likely was a digestive enzyme involved in food digestion.

### Spatial-temporal expression of *Ca*-chit mRNA in different developmental larvae

**Fig 5:**
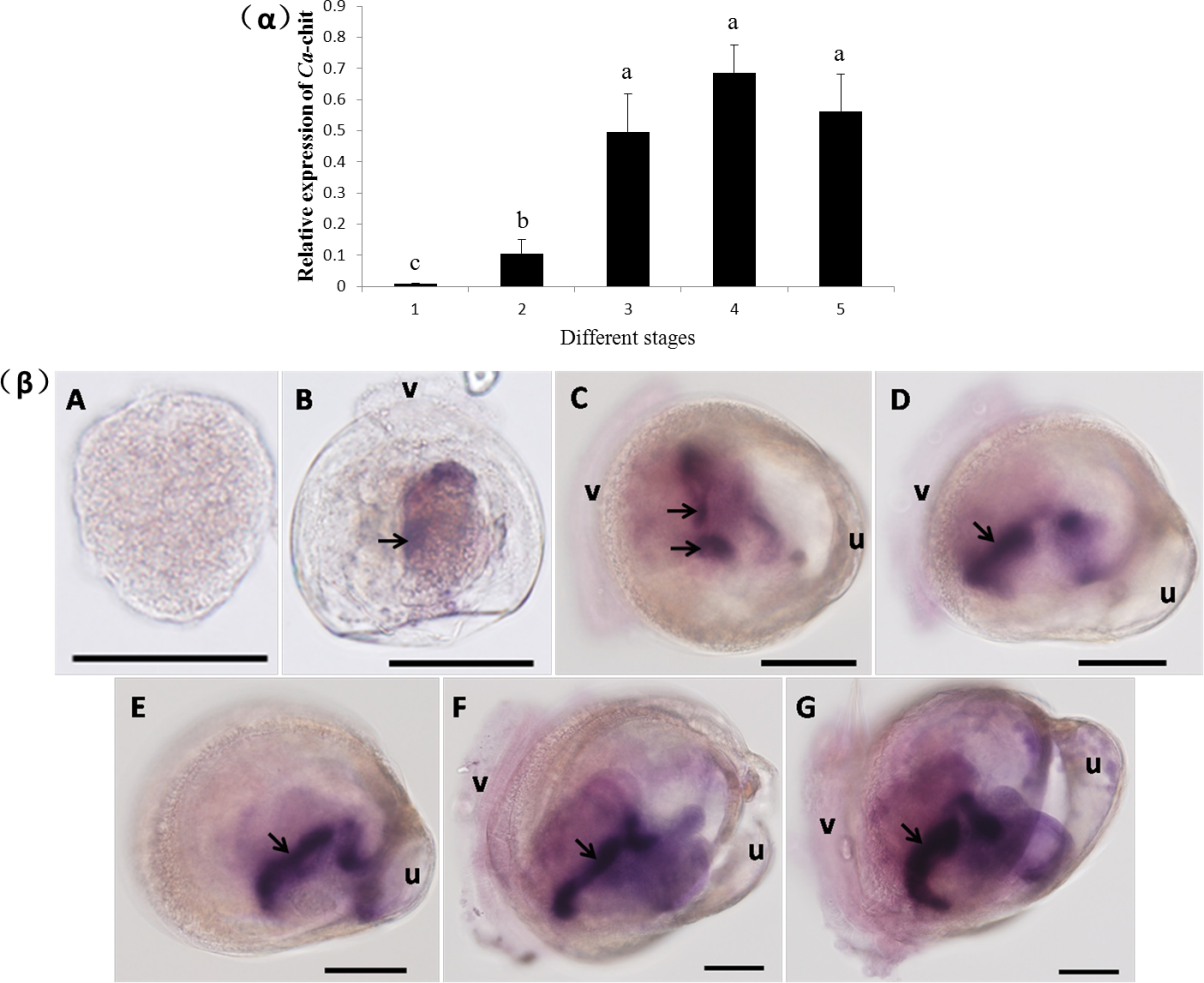
*Ca*-Chit mRNA expression profiles in the larvae from trochophore to umbo-veliger larvae by qRT-PCR (α) and whole mount in situ hybridization (β). Each bar represents the mean ± SD of three replicates from three oysters. Data with significant difference between each other at *P<0*.*05* are indicated by different letters. 1, A: trochophore no detectable signal; 2, B: D-veliger larvae signals in the visceral mass (arrow); 3, C, D and E: early stages of umbo-veliger signals in the visceral mass (arrow); 4, F: middle stage of umbo-veliger signals in the visceral mass (arrow); 5, G: later stage of umbo-veliger signals in the visceral mass (arrow); u: larval umbo; v: larval veliger. The scale of black bar is 50 um.

The expression pattern of *Ca*-chit in different developmental larvae was studied by the whole-mount in situ hybridization and qRT-PCR. As shown in Fig 5α, *Ca*-chit mRNA was highly expressed in umbo-veliger larvae. *Ca*-Chit mRNA levels was at least 5-fold more than the level in D-veliger larvae (*p<0*.*05*)。The data in Fig 5β showed that no *Ca*-chit mRNA was expressed in trochophore larvae, but in D-veliger larvae, *Ca*-chit expression began with the signal position located dispersedly in the visceral mass. When the larvae developed into the umbo-veliger stage, the site of *Ca*-chit signals was intensively located in part of the visceral mass. The clavate shape of the *Ca*-chit signal location lead us to believe that *Ca*-chit is exclusively expressed in the crystalline structure of the visceral mass.

### Adult oyster starvation experiment

**Fig 6:**
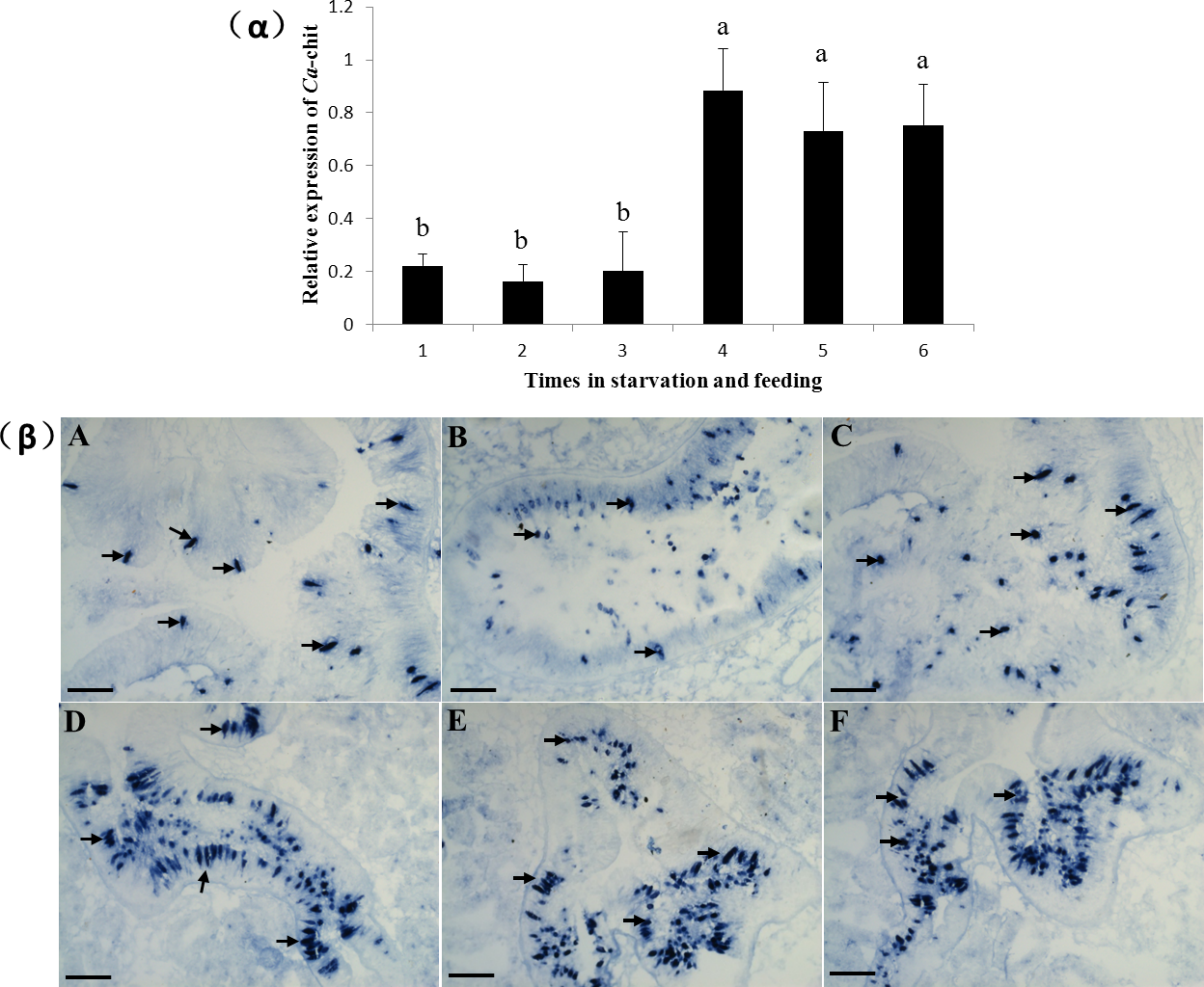
Expression analysis of *Ca*-Chit mRNA in the visceral mass of adult oyster after starvation and feeding challenge by qRT-PCR (α) and whole mount in situ hybridization (β). Each bar represents the mean ± SD of three replicates from three oysters. Data with significant difference between each other at *P<0*.*05* are indicated by different letters. 1, A: 6 day after starvation; 2, B: 7 day after starvation; 3, C: 2 h after feeding; 4, D: 9 h after feeding; 5, E: 2 day after feeding; 6,F: normal feeding oyster. Signals were labelled with arrow. The scale of black bar is 100 um.

As shown in Fig 6α, during the Adult oyster starvation experiment, the mRNA of *Ca*-chit was expressed at a low level, and then after cultured with algae and soy milk. *Ca*-chit expression levels began to rise at the 2th h, and became highly expressed in 9th h which expressed level is 4-fold more than the level at the 2th h and 5-fold more than the level before feeding. After 48 hours with the feed treatment, *Ca*-chit expression was reduced by 17.3% compared with the level at 9th h but still 3-fold more than that at the 2th h. The results in situ hybridization showed in Fig 6β were most consistent with the data of qRT-PCR, *Ca*-chit mRNA was localized in the apical lamina of digestive lumens and expressed in a low level after starvation stimulation, but strong sensitivity of detection was found in the digestive lumens after cultured with algae and soy milk.

## Discussion

In this study, the cloned sequence encoding chitinase from the oyster *Crassostrea angulata* was characterized, leading to the first report being done on their enzymatic role in the digestive system. Multiplying the alignment of *Ca*-Chit with some of these homologous proteins revealed that the primary structure of *Ca*-Chit is conserved with that of other species while possessing two putative catalytically active domains, which is different due to the chitinase playing a role in the immune system [17] and the chitinase-like protein [18,28]. The conserved catalytically active domain of *Ca*-Chit is an eight-stranded alpha/beta barrel fold and belongs to the family, 18 glycosyl hydrolases [29]. Each catalytically active domain has an active centre, which is located in the groove at the enzyme surface. The nine conserved amino acids are supposed to be involved in the enzymatic hydrolysis of glycosidic bonds [30]. Following the second catalytically active domain is a threonine-rich region. In this region, the sequence possesses 37% threonine. The high abundance of threonine-rich regions is predicted to be O-glycosylated in *Ca*-chit protein [31]. In terms of position, the threonine-rich region likely functions as an isolation area to separate the catalytic domain and the transmembrane domain. It also may serve to prevent proteolysis or aid in the secretion of the chitinase [32].

*Ca*-chit has the highest identity with Chitinase3 from the subspecies *C*.*gigas*. The protein composition of *Ca*-chit and chitinase3 from *C*.*angulata* are both more than 1000 amino acids with two active sites, but the protein composition of chitinase1 is less than 500 amino acids with one active site in *C*. *gigas*. While the *Ca*-chit identity with chitinase3 from *C*. *gigas* is 67.58%, but the identity with chitinase1 from *C*.*gigas* is only 11.89%. Phylogenetic tree analysis also demonstrated that it is relatively more closely to molluscan chitinase3 (Fig 3). Based on these results we speculate that the *Ca*-chit is part of the chitinase3 family.

Generally many of the other chitinases of family 18 were reported to be secreted in the extracellular space, or accumulate intracellularly as soluble enzymes [32, 33]. In our study, at the C- terminus of the *Ca*-Chit protein there is a transmembrane domain which is constituted of 23 amino acids and predicted to constitute a membrane anchor domain. In previous studies, two transmembrane domains were predicted by computer modeling [34], and later, the first chitinase sequence of family 18 to contain a transmembrane domain was characterized from *Crossostrea gigas* [17], but their functions were both shown to play a role in the immune processes.

Analysis of mRNA distribution in adult tissues reveals that *Ca*-chit is expressed highly in the visceral mass and less in other tissues. In previous studies, the chitinase of family 18 was found to express mainly in haemocytes and concluded that chitinase play an important role in the immune processes of invertebrates [17]. In present studies, *Ca*-chit is very weakly expressed in haemocytes, implicating it is not involved in any of the immune system’s processes. Visceral mass, which is considered to be the main digestive tissue in mollusks [35], expressed *Ca*-chit highly. Additionally, the starvation experiment indicated that *Ca*-chit mRNA was up-regulated in visceral mass by stimulation of feeding after starvation, and the results in Fig 6β show that *Ca*-chit mRNA concentrate on the apical lamina of digestive lumens. These observations imply that *Ca*-chit is likely involved in the digestive processes, which make this the first report to conclude that the molecular level of chitinase, of family 18, is involved in the digestive system of mollusks.

In-situ hybridization analysis of *Ca*-chit expression revealed that no *Ca*-chit mRNA was expressed in trochophore larvae, until the D-veliger larvae *Ca*-chit mRNA started to express at the location of the visceral mass. This is consistent with the D-veliger larvae at the stage which the larvae start feeding [36]. Visceral mass consists of the stomach, mid-gut, style sac, intestine, and part of the rectum [37]. At early stages of D-veliger, some parts of the visceral mass have not fully developed, which is likely the reasoning for why the signal position was located dispersedly in the visceral mass of the D-veliger larvae. Afterwards, when the larvae develop to the umbo-veliger stage, the expression site of *Ca*-chit was intensively located in part of the visceral mass, indicating that the visceral mass may have fulfilled differentiation. During the middle stage of umbo-veliger larvae, the expression location was larger. After the umbo-veliger larvae develop to a later period, the signal site of *Ca*-chit was shown clearly and formed into a ‘clavate’ shape. In previous studies, specific activity of chitinase was present in the *Crassostrea virginica* crystalline structures [16] and the *Mytilus edulis* contained the highest concentrations of chitinase found in the crystalline structure and in the digestive gland [19]. In filter-feeding bivalves, the crystalline structure is an elongated rod of solidified mucus, which projects into the stomach from an associated sac, and extends along the neutral arms of the visceral mass; an important part of the digestive system [38,39]. In conclusion, the clavate shape of *Ca*-chit expression lead us to speculate that *Ca*-chit was exclusively expressed in the crystalline structures of the visceral mass in order to fulfill an important function as a digestive enzyme in mollusks.

In conclusion, we have identified a full cDNA encoding chitinase of family 18 from the oyster *Crassostrea angulata*, named *Ca*-chit. *Ca*-chit mRNA was remarkably expressed in the crystalline structures of the visceral mass and was regulated by feeding, indicating that *Ca*-chit is probably an important digestive enzyme in the visceral mass of oysters. This marks the first time that chitinase of family 18 to be characterized as a digestive enzyme at the molecular level in bivalve mollusks.
